# Comparative Performance of Quantitative and Qualitative Magnetic Resonance Imaging Metrics in Primary Sclerosing Cholangitis

**DOI:** 10.1016/j.gastha.2022.01.003

**Published:** 2022-03-30

**Authors:** John E. Eaton, Christopher L. Welle, Hannah Monahan, Ali Dean Tahboub Amawi, Ilkay Idilman, William S. Harmsen, Bogdan Dzyubak, Elizabeth W. Beiermann, Zeinab Bakhshi, Gregory J. Gores, Nicholas F. LaRusso, Andrea A. Gossard, Konstantinos N. Lazaridis, Sudhakar K. Venkatesh

**Affiliations:** 1 Division of Gastroenterology & Hepatology, Mayo Clinic, Rochester, Minnesota; 2 Department of Radiology, Mayo Clinic, Rochester, Minnesota; 3 Division of Biomedical Statistics and Informatics, Mayo Clinic, Rochester, Minnesota; 4 Department of Internal Medicine, Mayo Clinic, Rochester, Minnesota

**Keywords:** Biomarker, Prognosis, Elastography, Spleen

## Abstract

**Background and Aims:**

Several quantitative and qualitative magnetic resonance imaging (MRI) metrics have been reported to predict outcomes among those with primary sclerosing cholangitis (PSC). We aimed to compare the reproducibility and prognostic performances of MRI biomarkers and examine if combining these measurements adds value.

**Methods:**

We performed a retrospective review of 388 patients with PSC who underwent a magnetic resonance elastography and magnetic resonance cholangiopancreatography. Liver stiffness (LS) was determined by validated automated software, whereas spleen volume was calculated by semiautomated software, and radiologists manually determined the ANALI scores. The primary endpoint was hepatic decompensation.

**Results:**

LS and spleen volume values had perfect and near-perfect agreement (intraclass correlation coefficient of 1.00 and 0.9996, respectively), whereas ANALI with and without gadolinium had a moderate inter-rater agreement between 3 radiologists (kappa = 0.42–0.54 and 0.46–0.57, respectively). As a continuous variable, LS alone was the best predictor of hepatic decompensation (concordance score = 0.90; 95% confidence interval, 0.87–0.93). A quantitative-only MRI model [LS (>4.70 kPa = 2 or ≤4.70 kPa = 0) + spleen volume (>600 mm^3^ = 1 or ≤600 mm^3^ = 0)] had the optimal reproducibility and performance (concordance score = 0.85; 95% confidence interval = 0.80–0.89) and enabled patient risk stratification by estimating the 5-year incidence of hepatic decompensation: 7.49%, 44.50%, 70.00%, and 91.30% (score 0–3).

**Conclusion:**

Quantitative MRI markers of fibrosis and portal hypertension generated by automated and semiautomated software are highly reproducible. LS is the single best imaging predictor of hepatic decompensation. However, a quantitative MRI score using LS and spleen volume is well suited to risk stratify those with PSC.

## Introduction

Primary sclerosing cholangitis (PSC) is a chronic cholestatic liver disorder characterized by inflammation and fibrosis of the intrahepatic and or extrahepatic bile ducts that can lead to cirrhosis and complications related to portal hypertension.^1^ It is a rare condition with a heterogeneous disease course which lacks an effective medical therapy. For these reasons, biomarkers which can predict outcomes are important in routine clinical care and in the conduct of therapeutic clinical trials.^2^

Information generated from magnetic resonance imaging (MRI) serves as an important PSC biomarker. Magnetic resonance cholangiopancreatography (MRCP) is the diagnostic test of choice for PSC and is used in the longitudinal follow-up to monitor for disease-related complications. It can provide a detailed assessment of the biliary tree, hepatic parenchyma, and surrounding structures.^3^ Quantitative features such as measurement of liver stiffness (LS) through magnetic resonance elastography (MRE) can be obtained concomitantly with a MRCP without adding a significant amount of time to the examination. MRE-derived LS values are associated with fibrosis stages and can predict hepatic decompensation.^4^, ^5^, ^6^ Spleen volume and length, surrogates for portal hypertension, can be quantified using MRI and may offer prognostic value.^7^, ^8^, ^9^ Qualitative MRI-derived markers have also been studied. For example, the ANALI score (a term derived from the names of the creators) with and without gadolinium (GAD) uses radiologist-gathered features concerning the appearance of the biliary tree, hepatic parenchyma, and findings suggestive of portal hypertension.^10^ A higher ANALI score (with or without GAD) has been associated with adverse clinical outcomes.^11^^,^^12^

Both quantitative and qualitative approaches offer their own unique advantages and disadvantages. For example, automated software and semiautomated software have the potential to provide objective measurements that are more reproducible and generalizable when compared with subjective assessments obtained by humans. Indeed, the inter-rater agreement between radiologists and bile duct changes visualized on MRCP is poor.^13^ Moreover, quantitative approaches can assess imaging features and biomechanical properties unseen by the human eye. Conversely, qualitative approaches can examine features of known significance that are not readily assessed by automated methods and parlay the benefits of clinical expertise. To date, the reproducibility and prognostic performances of quantitative and qualitative MRI biomarkers to predict outcomes in those with PSC have not been compared. Moreover, it remains unclear if combining multiple MRI features would offer an advantage over any single imaging biomarker. Consequently, we assessed the reproducibility of existing quantitative and qualitative MRI predictive tools, compared their prognostic performances, and investigated whether a composite MRI biomarker has merit.

## Materials and Methods

### Patients

This study was approved by the Institutional Review Board at Mayo Clinic, Rochester, MN, and conforms to the ethical guidelines of the 1975 Declaration of Helsinki. A retrospective review was conducted between January 1, 2007 and May 1, 2018. Patients were included if they had diagnostic features of large-duct PSC as previously defined and had 1 MRE with MRCP with GAD at our institution.^14^ MRE was performed along with MRCP for cholangiocarcinoma (CCA) screening. Individuals who receive their regular care at our institution have laboratory tests every 3–6 months and an annual MRCP for CCA screening. Patients were excluded if they had small-duct PSC or a prior history of CCA, hepatic decompensation, prior liver transplant, or other hepatobiliary surgery (except cholecystectomy) before their imaging study. A subset of this cohort (n = 266) was included in an earlier study, and substantive additional clinical and imaging data were collected on these subjects.^4^

### Data Collection and Key Definitions

Laboratory data, including PSC risk estimate tool (PRESTO), the revised Mayo PSC risk, and model for end-stage liver disease (MELD) scores, were collected at the time of imaging. Serum alkaline phosphatase was divided by the upper limit of normal (ULN).^15^, ^16^, ^17^ Hepatic decompensation was defined by the development of ascites, variceal hemorrhage, or hepatic encephalopathy.^4^^,^^5^^,^^18^ Patients with ascites detected on imaging were counted as having hepatic decompensation even if a paracentesis was not required. CCA was diagnosed based on typical imaging features or cytology or biopsy positive for adenocarcinoma.^3^

MRE examinations were performed as previously described, and LS values were expressed in kilopascals (kPa).^4^ LS was quantified using an automated validated software program called automatic liver elasticity calculation which has been studied in PSC and other chronic liver diseases.^4^^,^^19^, ^20^, ^21^ Spleen volume was measured by 2 independent reviewers using a semiautomated 3-dimensional volumetry option available on a standard picture archival and communication system (Visage Imaging GmbH) and expressed in mm^3^. Spleen length was assessed by 2 readers using the standard caliper method and measuring the longest length of the spleen in any direction.

As previously reported, the ANALI score without GAD can range from 0 to 5, and the formula is as follows: [1 × intrahepatic bile duct dilation (IHBD)] + [2 × dysmorphy] + [1 × portal hypertension]. Similarly, the ANALI score with GAD may range from 0 to 2, and the formula is [1 × dysmorphy] + [1 × parenchymal enhancement heterogeneity]. For the purposes of calculating the ANALI scores, we used the previously reported component definitions described by the score’s creators. For example, IHBD was scored 2 if dilation was ≥5 mm, 1 if equal to 4 mm, and 0 if ≤3 mm.^10^, ^11^, ^12^ Before the conduct of the study, the lead radiologist for this study communicated with the developers of the ANALI score to ensure consistency and to minimize reporting errors. Three radiology reviewers, blinded to the clinical information, examined all images to determine the ANALI score with and without GAD and their individual components. All radiologists work at a high-volume PSC-MRI center. Reviewer 1 is an abdominal radiologist with an estimated experience >6000 MRCPs (>1500 with PSC); reviewer 2 is an abdominal radiologist with an estimated experience of >1000 MRCPs (>500 with PSC), whereas reviewer 3 is an abdominal radiology fellow with an estimated experience of >200 MRCPs (>50 with PSC). The ANALI scores assigned by reviewer 1 were used to assess their prognostic performance.

### Statistical Analysis

Statistical analysis was performed with JMP and SAS software (SAS Institute; Cary, NC). All tests were 2-sided with a level of significance of *P* < .05. Categorical data were compared using the Pearson chi-squared test, and continuous variables were compared using the nonparametric Wilcoxon test. Categorical data are presented as numbers (percentages), whereas continuous variables are expressed as medians and interquartile ranges unless otherwise stated. The Spearman correlation coefficient (r_s_) was used to measure association between variables.

The intraclass correlation coefficient (ICC) quantified reproducibility of LS with repeated measurements and spleen volume and length between 2 radiologists. The inter-rater variability between 3 radiologists and assigning ANALI scores and the score components was determined by the kappa statistic.

The primary endpoint was the development of hepatic decompensation. Patients who did not develop the endpoint were censored at the time of liver transplantation, CCA diagnosis, death, or date of the last follow-up (whichever one occurred earlier). The secondary endpoint was the development of hepatic decompensation, liver transplant, or death (all-cause). In this analysis, patients were censored at the date of the last follow-up. Cox proportional hazards regression analysis was used in both univariable and multivariable analyses to examine associations between covariates and the primary endpoint, and the results were expressed as hazard ratios and 95% confidence intervals (CIs). A multivariable model was created using the backward selection method retaining those variables with a significance level less than 0.05 process. The prognostic performance of selected imaging variables was examined in the entire cohort and key patient subgroups (portal hypertension present or absent; total bilirubin >2.0 mg/dL or ≤2.0 mg/dL; serum alkaline phosphatase ≤1.5 × ULN or >1.5 × ULN).

The discriminative ability of MRI variables to categorize individuals at various risks for developing hepatic decompensation was assessed with the concordance score from the Cox model. MRI variables were treated as a continuous variable and then as dichotomous variables, whereby the optimal cutoff to predict hepatic decompensation was selected by using the criteria of Contal and O’Quigley.^22^ We examined both continuous and dichotomized covariates for several reasons. Dichotomizing continuous variables can simplify data interpretation and enable risk stratification. However, it is known this can attenuate the predictive power of a continuous variable.^23^

**Figure 1:**
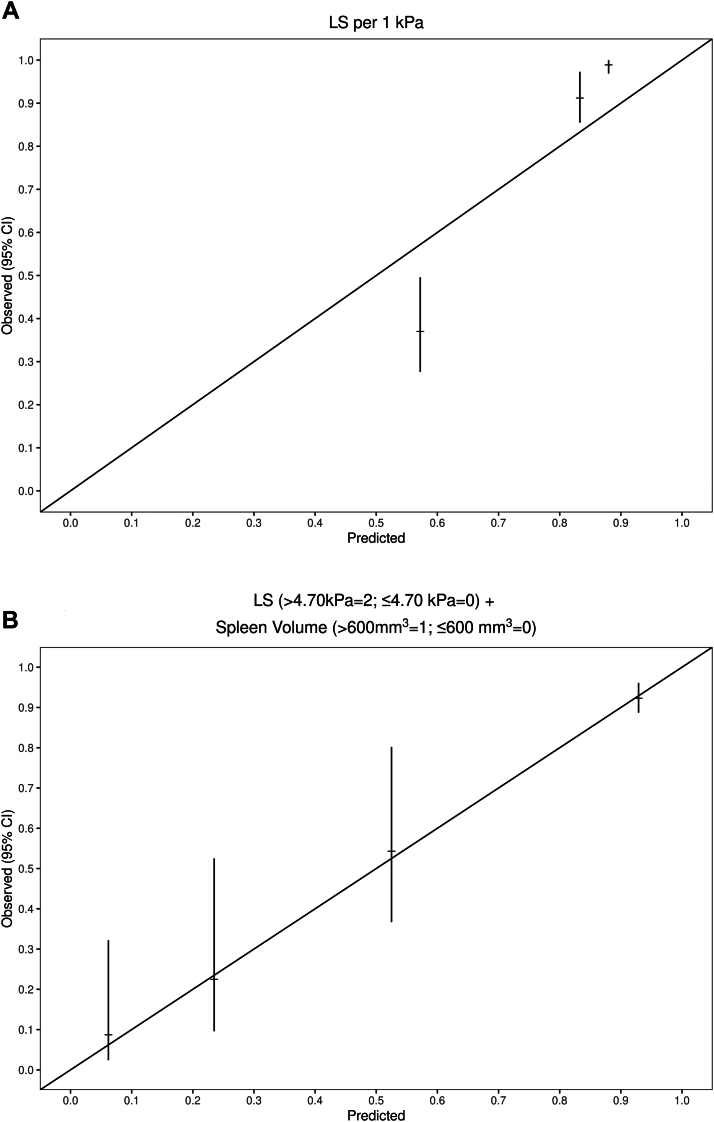
Calibration plots illustrating probability of observed vs predict events of hepatic decompensation. (A) Liver stiffness (continuous variable). (B) Quantitative MRI model: Liver stiffness (>4.70 kPa = 2 or ≤4.70 kPa = 0) + spleen volume (>600 mm^3^ = 1 or ≤600 mm^3^ = 0). In panel A, the 3 categories correspond to the tertiles of the LS distribution for the cohort, whereas in panel B, they correspond to a score from the model (score 0–4). Horizontal bars represent the probability point estimate, and vertical bars indicate the 95% confidence interval. CI, confidence interval; LS, liver stiffness.

The ability of select covariates to accurately predict the endpoint of interest across various risk groups (ie, calibration) was assessed by using predicted probabilities at 5 years which were grouped into quintiles or tertiles to have approximately equal numbers of patients in each. Subsequently, the mean predicted and observed probability of hepatic decompensation in each risk group and the 95% CIs for the observed probability were identified and illustrated graphically (Figure 1).

## Results

### Patients and Imaging

Three hundred eighty-eight patients with PSC were included in this study and followed for a median of 5.10 years (2.50–6.10) years. No patients were excluded owing to suboptimal imaging. The baseline features at the time of imaging are shown in Table 1.

**Table 1:** Baseline Features

Age (y)	44.37 (31.61–57.96)
Female	34.02% (132/388)
PSC duration (y)	5.29 (1.01–11.94)
IBD present	78.87% (306/388)
Portal hypertension present	12.37% (48/388)
SAP/ULN	1.62 (0.89–2.94)
AST	48.00 (29.00–81.75)
Total bilirubin (mg/dL)	0.80 (0.50–1.30)
Platelets (×10^9^/L)	244.00 (196.00–300.00)
MELD score	7.60 (5.60–9.80)
Mayo PSC risk score	−0.50 (−1.10 to 0.30)
PRESTO (%)	4.60 (3.20–10.80)
LS (kPa)	2.90 (2.40–3.90)
Spleen length (mm)	108.50 (92.50–123.60)
Spleen volume (mm^3^)	306.00 (195.30–470.00)
ANALI-GAD	1.00 (1.00–2.00)
0	39.4% (153/388)
1	33.5% (130/388)
2	27.1% (105/388)
ANALI-no GAD	2.00 (1.00–2.00)
0	26.5% (103/388)
1	15.7% (61/388)
2	27.8% (108/388)
3	10.8% (42/388)
4	12.9% (50/388)
5	6.2% (24/388)

AST, aspartate aminotransferase; GAD, gadolinium; IBD, inflammatory bowel disease; LS, liver stiffness; MELD, model for end-stage liver disease; PRESTO, PSC risk estimate tool; PSC, primary sclerosing cholangitis; SAP, serum alkaline phosphatase; ULN, upper limit of normal.

Hepatic decompensation developed in 77 subjects (ascites n = 69; variceal bleeding n = 15, hepatic encephalopathy n = 20). CCA developed in 18 individuals after their baseline imaging. Thirty-five participants underwent liver transplantation (indications: hepatic decompensation alone n = 23; CCA n = 6; hepatocellular carcinoma n = 1; refractory pruritus or ascending cholangitis n = 5), whereas 23 died (CCA n = 5; liver failure n = 5; infection n = 3; colorectal cancer n = 1; unknown n = 1; other non-PSC related n = 8).

The correlations between baseline imaging and biochemical parameters are shown in Table A1. Compared with the ANALI scores, LS had a stronger correlation with MELD (r_s_ = 0.38), total bilirubin (r_s_ = 0.46), Mayo PSC risk score (r_s_ = 0.65), and PRESTO (r_s_ = 0.61). Intrahepatic bile duct dilation did not correlate well with LS (r_s_ = 0.23), spleen volume (r_s_ = 0.11), total bilirubin (r_s_ = 0.13), alkaline phosphatase (r_s_ = 0.04), and features of portal hypertension (r_s_ = 0.17).

### Reproducibility of Quantitative vs Qualitative MRI Features

Software was able to demonstrate perfect reproducibility on repeated measurements of LS (ICC = 1.00) and nearly perfect reproducibility for spleen volume (ICC = 0.9996; 95% CI, 0.99–1.00). The reproducibility of spleen length was almost perfect: ICC = 0.88; 95% CI, 0.80–0.93.

In contrast, the reproducibility of ANALI (kappa = 0.42–0.57) and its components was moderate when compared across 3 radiologists (Table 2). The agreement between the 2 most experienced radiologists was slightly higher when compared with the radiologist with the least experience (reviewer 3) (Table 2). The reviewers had the strongest agreement on the presence of portal hypertension (kappa = 0.61–0.70) and the weakest agreement on the presence of parenchymal enhancement heterogeneity (kappa = 0.40–0.56).

**Table 2:** Inter-rater Variability of ANALI Scores

Reviewers	ANALI-GAD total	ANALI-no GAD total	Dysmorphy	PHTN	IHBD	PEH
Kappa (95% CI)						
1 vs 2	0.54 (0.47–0.61)	0.56 (0.50–0.61)	0.58 (0.50–0.66)	0.70 (0.61–0.80)	0.59 (0.53–0.66)	0.56 (0.46–0.65)
1 vs 3	0.42 (0.36–0.49)	0.46 (0.40–0.52)	0.44 (0.36–0.52)	0.61 (0.50–0.73)	0.48 (0.42–0.54)	0.48 (0.40–0.56)
2 vs 3	0.47 (0.40–0.53)	0.57 (0.52–0.63)	0.60 (0.52–0.67)	0.63 (0.52–0.74)	0.61 (0.55–0.67)	0.40 (0.32–0.47)

GAD, gadolinium; IHBD, intrahepatic bile duct dilation; PEH, parenchymal enhancement heterogeneity; PHTN, portal hypertension.

### Prognostic Significance of Individual Quantitative and Qualitative Imaging Parameters

Table 3 illustrates the covariates examined in the univariate analysis to predict hepatic decompensation. LS as a continuous variable was the single best predictor of hepatic decompensation (concordance score = 0.90; 95% CI, 0.87–0.93), and it continued to perform well in key patient subgroups (Table A2). Figure 1A illustrates the calibration of LS to predict hepatic decompensation across risk groups. By comparison, ANALI with and without GAD had respective concordance scores of 0.75 and 0.79. Interestingly, increasing IHBD (component of ANALI without GAD) had a marginal ability to predict hepatic decompensation (concordance score = 0.60; 95% CI, 0.54–0.67).

**Table 3:** Imaging Predictors of Hepatic Decompensation

Imaging variables	HR (95% CI)	*P* value^b^	Concordance (95% CI)
Continuous variable^a^			
LS per 1 kPa	1.61 (1.50–1.74)	<.001	0.90 (0.87–0.93)
ANALI-GAD per 1 unit	3.47 (2.49–4.84)	<.001	0.75 (0.70–0.79)
ANALI-no GAD per 1 unit	1.87 (1.60–2.17)	<.001	0.79 (0.75–0.83)
Spleen length per 1 mm	1.04 (1.03–1.05)	<.001	0.76 (0.71–0.81)
Spleen volume per 1000 mm^3^	9.00 (5.96–13.61)	<.001	0.79 (0.74–0.85)
Dichotomous variables^a^			
LS >4.70 kPa	15.10 (9.40–24.26)	<.001	0.78 (0.73–0.83)
ANALI-GAD >1	5.59 (3.52–8.87)	<.001	0.71 (0.68–0.76)
ANALI-no GAD >2	6.25 (3.86–10.14)	<.001	0.73 (0.68–0.78)
Spleen length >140 mm	8.45 (5.22–13.67)	<.001	0.66 (0.61–0.72)
Spleen volume >600 (mm^3^)	7.74 (4.90–12.23)	<.001	0.71 (0.65–0.76)
Multivariable models			
Dichotomous quantitative and qualitative			0.88 (0.85–0.92)
LS (>4.70 kPa = 2; ≤4.70 kPa = 0) + ANALI-no GAD (>2 units = 1; ≤2 units = 0) + Spleen volume (>600 mm^3^ = 1; ≤600 mm^3^ = 0)^c^			
Score 0	(Reference)		
Score 1	9.90 (3.93–24.94)	<.001	
Score 2	24.67 (9.08–66.99)	<.001	
Score 3	57.69 (22.86–145.61)	<.001	
Score 4	76.63 (30.64–191.64)	<.001	
Dichotomous quantitative only			0.85 (0.80–0.89)
LS (>4.70 kPa = 2; ≤4.70 kPa = 0) + Spleen volume (>600 mm^3^ = 1; ≤600 mm^3^ = 0)^c^			
Score 0	(Reference)		
Score 1	8.80 (4.20–18.46)	<.001	
Score 2	19.28 (10.12–36.73)	<.001	
Score 3	34.91 (18.40–66.21)	<.001	

CI, confidence interval; GAD, gadolinium; HR, hazard ratio; LS, liver stiffness.

a Unadjusted variables.

b Cox proportional hazards regression.

c Point estimates for each score shown are compared with score 0 (reference group). Discriminative ability of each additional score relative to each other is shown in Table A5.

The addition of other imaging variables including the ANALI scores did not enhance the performance of LS as a continuous variable (Table A3). Similarly, LS remained independently associated with hepatic decompensation after adjusting for MELD, Mayo PSC risk, and PRESTO scores (Table A4).

The optimal cutoff to predict hepatic decompensation was determined for LS, ANALI scores, and spleen volume and length (Table 3). As anticipated, dichotomization tended to lower the predictive power of the individual covariates. For example, when LS is dichotomized using the optimal cutoff (4.70 kPa) to predict hepatic decompensation, its performance becomes attenuated (concordance score = 0.78; 95% CI, 0.73–0.83).

### Prognostic Performance of Composite MRI Risk Scores

To enhance the predictive performance of individual dichotomous variables, we examined them (LS, ANALI with and without GAD, and spleen length and volume) through a parsimonious model using backward selection. This noted that LS, ANALI without GAD, and spleen volume were the most significant dichotomous predictors of hepatic decompensation and generated a weighted quantitative and qualitative MRI model: LS (>4.70 kPa = 2 or ≤4.70 kPa = 0) + ANALI without GAD (>2 = 1 or ≤2 = 0) + spleen volume (>600 mm^3^ = 1 or ≤600 mm^3^ = 0). The performance of this composite model to predict hepatic decompensation was excellent (concordance score = 0.89; 95% CI, 0.85–0.92) (Table 3). However, a score of 4 did not appear to differ from a score of 3 in its ability to predict hepatic decompensation (Table A5).

**Figure 2:**
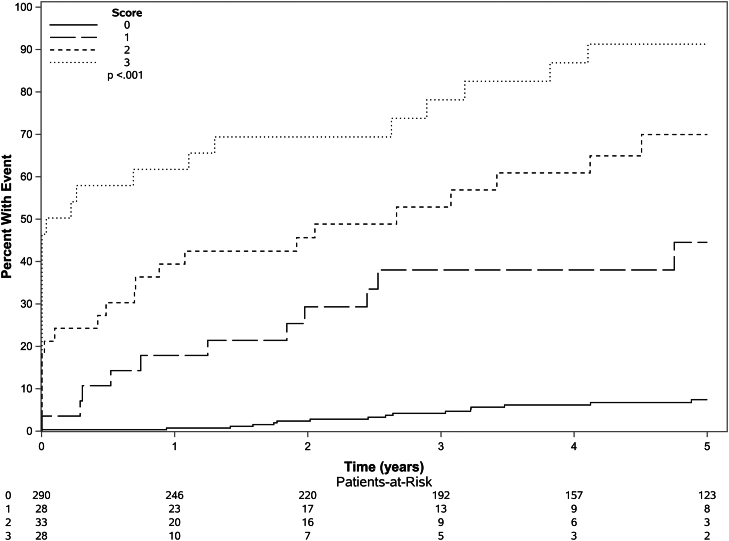
Cumulative incidence of hepatic decompensation across risk groups. Score 0–3 determined by the quantitative MRI model: Liver stiffness (>4.70 kPa = 2 or ≤4.70 kPa = 0) + spleen volume (>600 mm^3^ = 1 or ≤600 mm^3^ = 0).

**Figure A1:**
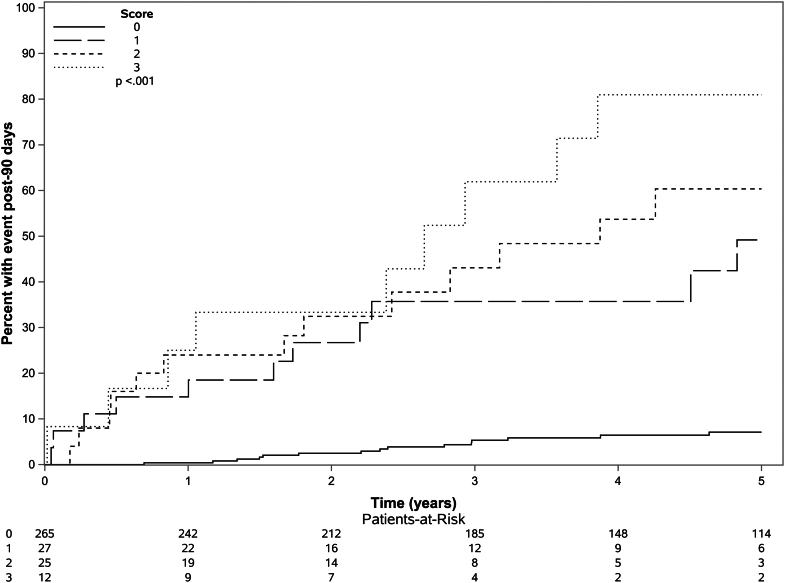
Cumulative incidence of hepatic decompensation across risk groups after starting 90 days after baseline image. Score 0–3 determined by Quantitative MRI model: Liver stiffness (>4.70 kPa = 2 or ≤4.70 kPa = 0) + spleen volume (>600 mm^3^ = 1 or ≤600 mm^3^ = 0).

Recognizing the limited contribution of the ANALI score to the overall performance of the abovementioned model and its modest reproducibility (Table 2), we removed the ANALI variable to examine the characteristic of a weighted dichotomized bivariate MRI score that used only quantitative variables obtained through highly reproducible automated-semiautomated software: LS (>4.70 kPa = 2 or ≤4.70 kPa = 0) + spleen volume (>600 mm^3^ = 1 or ≤600 mm^3^ = 0) (Table 3). Notably, the performance of the quantitative model after the elimination of the ANALI score remained excellent (concordance score = 0.85; 95% CI, 0.80–0.89) and comparable with the performances of LS alone as a continuous variable and the combined quantitative and qualitative model which included the ANALI score (Table 3). This approach enabled patients to be risk stratified into 4 groups (score 0–3), which demonstrated a 5-year cumulative incidence (95% CI) of hepatic decompensation of 7.49% (4.60%–12.00%), 44.50% (28.00%–70.80%), 70.00% (54.50%–89.80%), and 91.30% (80.50%–100%), *P* < .001 (Figure 2). This model performed well in key patient subgroups (Table A2) and remained independently associated with hepatic decompensation after adjusting for MELD, Mayo PSC risk, and PRESTO scores (Table A4). Moreover, when compared with LS alone, this quantitative model appeared better calibrated to assess 5-year freedom from hepatic decompensation across various risk groups (Figure 1B). Furthermore, this model (unlike the combined qualitative and quantitative model) accurately discriminates outcomes between each group (score 0–3) (Table A5). In addition, this dichotomized composite model performed better than using the low (<4.5 kPa), medium (4.5–6.0 kPa), and high (>6.0 kPa) risk cutoffs we reported previously^4^: 0.79; 95% CI 0.74–0.84 vs concordance score 0.85; 95% CI, 0.80–0.89; *P* value = .01. When we only considered nonearly events and shifted the baseline to 3 months after the imaging study, this model continued to enable risk stratification (Figure A1) and had good discriminative ability (concordance score = 0.78; 95% CI, 0.72–0.85). Finally, this model was also able to discriminate those at higher risk liver transplant, death, or hepatic decompensation (concordance score = 0.80; 95% CI 0.76–0.85) (Table A6).

## Discussion

In this study, we present the largest cohort of patients with PSC who underwent MRCP and MRE imaging to date and performed a comparative assessment of quantitative (LS and spleen volume and length) and qualitative (ANALI scores with and without GAD) MRI features. This work has several key findings. First, quantitative MRI features have superior reproducibility when compared with their qualitative counterparts. Second, LS as a continuous variable is the single best imaging predictor of hepatic decompensation, and the addition of ANALI or other variables did not enhance its predictive performance. Moreover, bile duct dilation alone appears to be a poor predictor of hepatic decompensation. Third, dichotomizing continuous variables can aid in clinical interpretation and patient risk stratification, but this comes at the cost of lowering the predictive power. When risk stratification is desired, a simple quantitative MRI model using LS and spleen volume provided the best combination of reproducibility and performance to predict outcomes across various risk groups and may have enhanced calibration compared with LS alone.

It is important to have noninvasive and reproducible biomarkers for clinical practice and therapeutic trials.^2^ As demonstrated here, quantitative measurements can be obtained by software which can enhance reproducibility without requiring advanced expertise or significant time investment. Automatic liver elasticity calculation, an automated algorithm to measure LS, is able to bypass the inefficiencies and provider variations which may occur with LS values generated by the manual region of interest selection.^19^^,^^21^ Qualitative assessments can be time-consuming and subject to human reporting variation which may limit their generalizability and reproducibility. For example, the ANALI score without GAD requires specialized expertise by a radiologist that involves subtle measurements of the biliary tree and assessments which may be subjective (eg, the presence of dysmorphy). Hence, automated and semiautomated quantitative imaging biomarkers are well-suited to play an increasing role in disease detection and prediction of outcomes in patients with chronic liver diseases. Another example of a quantitative imaging technique is the MRI-derived proton density fat fraction. This approach has been used in early nonalcoholic fatty liver disease trials and is highly reproducible and accurate in measuring hepatic steatosis.^24^

LS alone as a continuous variable is the single most important imaging predictor of hepatic decompensation in those with PSC, and our findings reinforce earlier observations on the value of LS and outcome prediction.^4^^,^^5^^,^^9^^,^^12^^,^^25^ Combining imaging variables only became advantageous when LS was dichotomized to enable risk stratification. In fact, a prior study illustrated that combining dichotomized LS values measured by transient elastography and ANALI without GAD was able to predict a composite endpoint (liver-related death, hepatic decompensation, and liver transplant) in those with PSC.^12^ In contrast, we did not find that ANALI added a substantial advantage to either LS as a continuous variable or a dichotomized model using LS and spleen volume. There may be several explanations for this. First, transient elastography (rather than MRE) was used, and the authors did not examine the impact of ANALI with LS as a continuous variable or examine spleen volume. Although uncertain, it is plausible that spleen volume (compared with LS) may be less subject to transient changes that can occur with biliary obstruction. Compared with transient elastography, MRE samples a larger volume of the liver by a thousand-fold which may be advantageous in a patchy disease such as PSC.^4^ Moreover, MRE is less operator and patient dependent and has been shown to more accurately detect various stages of fibrosis in other chronic liver diseases.^26^^,^^27^ Second, Cazzagon et al also included endpoints which may not be mediated portal hypertension and advancing liver fibrosis (eg, death from CCA or ascending cholangitis), and the timing and indications for transplant can vary. The degree of bile duct dilation that is incorporated in the ANALI score does not correlate well with markers of advancing fibrosis and portal hypertension (Table A1) and poorly predicted hepatic decompensation. Hence, parenchymal features and markers of portal hypertension may be more relevant in the prediction of hepatic decompensation. Yet, qualitative biliary metrics are useful for the early detection of CCA,^28^ and the role of quantitative biliary metrics to predict and diagnose biliary cancer deserves further study. Indeed, it would be beneficial to have a portfolio of outcome-specific biomarkers given the diverse array of complications associated with PSC.

This study has several limitations. First, it was a retrospective study conducted at a single center. Yet, this cohort represents the largest assembly of patients with PSC who underwent any form of MRI which was systematically analyzed to identify prognostic features to date. In addition, the individual prognostic importance of LS measured by MRE and spleen volume has been demonstrated in other centers.^6^^,^^7^^,^^29^ However, it will be important to validate these findings. Second, we did not examine the relationship between changes in imaging over time and the development of adverse outcomes. Although changes in LS measured by MRE are associated with hepatic decompensation in PSC, it remains unclear if a change in the composite MRI model would similarly predict adverse events.^5^ Third, although MRE has been available for over a decade and has other advantages for those with PSC, it is not as prevalent as transient elastography particularly outside of North America.^4^ Consequently, it would be prudent to validate if combining spleen volume and LS measured by transient elastography has value.

In conclusion, our findings highlight the merits of quantitative MRI biomarkers to predict hepatic decompensation in those with PSC. Quantitative metrics such as LS and spleen volume generated by automated and semiautomated techniques are more reproducible than qualitative assessments. LS and spleen volume may facilitate risk stratification of patients with PSC in clinical practice and in clinical trials.

## Supplementary Materials

Table A1

Table A2

Table A3

Table A4

Table A5

Table A6
