# Supplementary material for: Comparative Performance of Quantitative and Qualitative Magnetic Resonance Imaging Metrics in Primary Sclerosing Cholangitis
Source: Gastro Hep Adv. 2022 Mar 30;1(3):287–95. doi: 10.1016/j.gastha.2022.01.003 (PMC11307538; doi:10.1016/j.gastha.2022.01.003)
Supplement: Table A1 [file mmc1.docx]

Supplementary Table 1. Correlations between imaging and biochemical parameters^a^

|  | LS |  | ANALI-GAD | ANALI-no GAD | Dysmorphy | PHTN | IHBD | PEH | Spleen Volume | Spleen  Length | MELD | Mayo PSC Risk | PRESTO | SAPxULN | AST | Total  Bilirubin | Platelets |
| --- | --- | --- | --- | --- | --- | --- | --- | --- | --- | --- | --- | --- | --- | --- | --- | --- | --- |
|  |  |  |  |  |  |  |  |  |  |  |  |  |  |  |  |  |  |
| LS | - |  | 0.48  <0.001 | 0.45  <0.001 | 0.38  <0.001 | 0.44  <0.001 | 0.23  <0.001 | 0.40  <0.001 | 0.53  <0.001 | 0.46  <0.001 | 0.38  <0.001 | 0.65  <0.001 | 0.61  <0.001 | 0.13  0.009 | 0.55  <0.001 | 0.46  <0.001 | -0.26  <0.001 |
|  |  |  |  |  |  |  |  |  |  |  |  |  |  |  |  |  |  |
| ANALI  -GAD |  |  | - | 0.73  <0.001 | 0.80  <0.001 | 0.39  <0.001 | 0.27  <0.001 | 0.83  <0.001 | 0.28  <0.001 | 0.28  <0.001 | 0.25  <0.001 | 0.43  <0.001 | 0.43  <0.001 | 0.03  0.58 | 0.33  <0.001 | 0.25  <0.001 | -0.23  <0.001 |
|  |  |  |  |  |  |  |  |  |  |  |  |  |  |  |  |  |  |
| ANALI  -no GAD |  |  |  | - | 0.80  <0.001 | 0.46  <0.001 | 0.71  <0.001 | 0.41  <0.001 | 0.30  <0.001 | 0.29  <0.001 | 0.28  <0.001 | 0.45  <0.001 | 0.43  <0.001 | 0.08  0.12 | 0.29  <0.001 | 0.25  <0.001 | -0.23  <0.001 |
|  |  |  |  |  |  |  |  |  |  |  |  |  |  |  |  |  |  |
| Dysmorphy |  |  |  |  | - | 0.34  <0.001 | 0.18  <0.001 | 0.34  <0.001 | 0.27  <0.001 | 0.30  <0.001 | 0.24  <0.001 | 0.30  <0.001 | 0.36  <0.001 | 0.07  0.19 | 0.17  <0.001 | 0.20  <0.001 | -0.29  <0.001 |
|  |  |  |  |  |  |  |  |  |  |  |  |  |  |  |  |  |  |
| PHTN |  |  |  |  |  | - | 0.17  <0.001 | 0.31  <0.001 | 0.40  <0.001 | 0.31  <0.001 | 0.26  <0.001 | 0.35  <0.001 | 0.38  <0.001 | 0.04  0.50 | 0.23  <0.001 | 0.32  <0.001 | -0.29  <0.001 |
|  |  |  |  |  |  |  |  |  |  |  |  |  |  |  |  |  |  |
| IHBD |  |  |  |  |  |  | - | 0.25  <0.001 | 0.11  0.03 | 0.07  0.15 | 0.14  0.04 | 0.34  <0.001 | 0.23  <0.001 | 0.04  0.39 | 0.23  <0.001 | 0.13  0.01 | 0.01  0.84 |
|  |  |  |  |  |  |  |  |  |  |  |  |  |  |  |  |  |  |
| PEH |  |  |  |  |  |  |  | - | 0.19  <0.001 | 0.18  <0.001 | 0.18  0.009 | 0.39  <0.001 | 0.35  <0.001 | -0.02  0.64 | 0.36  <0.001 | 0.21  <0.001 | -0.10  0.05 |
|  |  |  |  |  |  |  |  |  |  |  |  |  |  |  |  |  |  |
| Spleen Volume |  |  |  |  |  |  |  |  | - | 0.86  <0.001 | 0.29  <0.001 | 0.36  <0.001 | 0.42  <0.001 | 0.05  0.31 | 0.33  <0.001 | 0.41  <0.001 | -0.52  <0.001 |
|  |  |  |  |  |  |  |  |  |  |  |  |  |  |  |  |  |  |
| Spleen Length |  |  |  |  |  |  |  |  |  | - | 0.26  <0.001 | 0.31  <0.001 | 0.37  <0.001 | 0.03  0.59 | 0.25  <0.001 | 0.33  <0.001 | -0.53  <0.001 |
|  |  |  |  |  |  |  |  |  |  |  |  |  |  |  |  |  |  |
| MELD |  |  |  |  |  |  |  |  |  |  | - | 0.55  <0.001 | 0.56  <0.001 | 0.17  0.02 | 0.40  <0.001 | 0.60  <0.001 | -0.06  0.36 |
|  |  |  |  |  |  |  |  |  |  |  |  |  |  |  |  |  |  |
| Mayo PSC Risk |  |  |  |  |  |  |  |  |  |  |  | - | 0.77  <0.001 | 0.13  0.03 | 0.64  <0.001 | 0.67  <0.001 | -0.20  <0.001 |
|  |  |  |  |  |  |  |  |  |  |  |  |  |  |  |  |  |  |
| PRESTO |  |  |  |  |  |  |  |  |  |  |  |  |  | 0.11  0.04 | 0.61  <0.001 | 0.65  <0.001 | -0.38  <0.001 |
|  |  |  |  |  |  |  |  |  |  |  |  |  |  |  |  |  |  |
| SAP x ULN |  |  |  |  |  |  |  |  |  |  |  |  |  | - | 0.08  0.13 | -0.02  0.64 | -0.06  0.21 |
|  |  |  |  |  |  |  |  |  |  |  |  |  |  |  |  |  |  |
| AST |  |  |  |  |  |  |  |  |  |  |  |  |  |  | - | 0.48  <0.001 | -0.05  0.32 |
|  |  |  |  |  |  |  |  |  |  |  |  |  |  |  |  |  |  |
| Total Bilirubin |  |  |  |  |  |  |  |  |  |  |  |  |  |  |  | - | -0.22  <0.001 |
|  |  |  |  |  |  |  |  |  |  |  |  |  |  |  |  |  |  |
| Platelets |  |  |  |  |  |  |  |  |  |  |  |  |  |  |  |  | - |

^a^ Spearman correlation coefficient & (p value) shown above

Abbreviations: GAD (gadolinium); PHTN (portal hypertension); IHBD (intrahepatic bile duct dilation); PEH (parenchymal enhancement heterogeneity); MELD (model end stage liver disease); SAP (serum alkaline phosphatase); ULN (upper limit of normal); AST (aspartate aminotransferase).
