# Supplementary material for: Comparative Performance of Quantitative and Qualitative Magnetic Resonance Imaging Metrics in Primary Sclerosing Cholangitis
Source: Gastro Hep Adv. 2022 Mar 30;1(3):287–95. doi: 10.1016/j.gastha.2022.01.003 (PMC11307538; doi:10.1016/j.gastha.2022.01.003)
Supplement: Table A2 [file mmc2.docx]

Supplementary Table 2. Performance of imaging parameters in patient subgroups

|  | HR  (95% CI) | P value^a^ | Concordance  (95% CI) |
| --- | --- | --- | --- |
| Portal Hypertension Present |  |  |  |
| - LS per 1 kPa | 1.40 (1.29-1.53) | <0.001 | 0.82 (0.77-0.86) |
|  |  |  |  |
| - LS (> 4.70kPa=2; ≤ 4.70 kPa=0) +   ANALI-no GAD (>2 units=1; ≤2 units=0) +  Spleen Volume (>600 mm^3^=1; ≤600 mm^3^=0) per point | 1.92 (1.60-2.30) | <0.001 | 0.80 (0.40-0.85) |
|  |  |  |  |
| - LS (> 4.70kPa=2; ≤ 4.70 kPa=0) +   Spleen Volume (>600 mm^3^=1; ≤600 mm^3^=0) per point | 2.24 (1.79-2.81) | <0.001 | 0.79 (0.73-0.84) |
| Portal Hypertension Absent |  |  |  |
| - LS per 1 kPa | 2.00 (1.60-2.50) | <0.001 | 0.86 (0.74-0.97) |
|  |  |  |  |
| - LS (> 4.70kPa=2; ≤ 4.70 kPa=0) +   ANALI-no GAD (>2 units=1; ≤2 units=0) +  Spleen Volume (>600 mm^3^=1; ≤600 mm^3^=0) per point | 4.50 (2.99-6.77) | <0.001 | 0.89 (0.81-0.97) |
|  |  |  |  |
| - LS (> 4.70kPa=2; ≤ 4.70 kPa=0) +   Spleen Volume (>600 mm^3^=1; ≤600 mm^3^=0) per point | 5.50 (3.29-9.07) | <0.001 | 0.80 (0.68-0.92) |
| Total Bilirubin ≤ 2 mg/dL |  |  |  |
| - LS per 1 kPa | 1.72 (1.55-1.90) | <0.001 | 0.90 (0.85-0.94) |
|  |  |  |  |
| - LS (> 4.70kPa=2; ≤ 4.70 kPa=0) +   ANALI-no GAD (>2 units=1; ≤2 units=0) +  Spleen Volume (>600 mm^3^=1; ≤600 mm^3^=0) per point | 2.92 (2.40-3.56) | <0.001 | 0.90 (0.86-0.94) |
|  |  |  |  |
| - LS (> 4.70kPa=2; ≤ 4.70 kPa=0) +   Spleen Volume (>600 mm^3^=1; ≤600 mm^3^=0) per point | 3.74 (2.92-4.79) | <0.001 | 0.86 (0.80-0.92) |
| Total Bilirubin > 2.0 mg/dL |  |  |  |
| - LS per 1 kPa | 1.27 (1.11-1.45) | <0.001 | 0.77 (0.68-0.86) |
|  |  |  |  |
| - LS (> 4.70kPa=2; ≤ 4.70 kPa=0) +   ANALI-no GAD (>2 units=1; ≤2 units=0) +  Spleen Volume (>600 mm^3^=1; ≤600 mm^3^=0) per point | 1.55 (1.19-2.02) | 0.001 | 0.74 (0.65-0.83) |
|  |  |  |  |
| - LS (> 4.70kPa=2; ≤ 4.70 kPa=0) +   Spleen Volume (>600 mm^3^=1; ≤600 mm^3^=0) per point | 1.73 (1.25-2.39) | <0.001 | 0.73 (0.64-0.83) |
| Serum Alkaline Phosphatase ≤ 1.5 x upper limit of normal |  |  |  |
| - LS per 1 kPa | 3.48 (2.28-5.30) | <0.001 | 0.98 (0.96-1.00) |
|  |  |  |  |
| - LS (> 4.70kPa=2; ≤ 4.70 kPa=0) +   ANALI-no GAD (>2 units=1; ≤2 units=0) +  Spleen Volume (>600 mm^3^=1; ≤600 mm^3^=0) per point | 5.77 (3.03-10.96) | <0.001 | 0.94 (0.83-1.0) |
|  |  |  |  |
| - LS (> 4.70kPa=2; ≤ 4.70 kPa=0) +   Spleen Volume (>600 mm^3^=1; ≤600 mm^3^=0) per point | 7.16 (3.57-14.36) | <0.001 | 0.94 (0.85-1.0) |
| Serum Alkaline Phosphatase > 1.5 x upper limit of normal |  |  |  |
| - LS per 1 kPa | 1.50 (1.37-1.63) | <0.001 | 0.84 (0.80-0.88) |
|  |  |  |  |
| - LS (> 4.70kPa=2; ≤ 4.70 kPa=0) +   ANALI-no GAD (>2 units=1; ≤2 units=0) +  Spleen Volume (>600 mm^3^=1; ≤600 mm^3^=0) per point | 2.10 (1.79-2.46) | <0.001 | 0.85 (0.80-0.89) |
|  |  |  |  |
| - LS (> 4.70kPa=2; ≤ 4.70 kPa=0) +   Spleen Volume (>600 mm^3^=1; ≤600 mm^3^=0) per point | 2.46 (2.01-3.01) | <0.001 | 0.81 (0.75-0.86) |
|  |  |  |  |

^a^ Cox proportional hazards regression

Abbreviations: LS (liver stiffness); GAD (gadolinium)
