# Supplementary material for: Comparative Performance of Quantitative and Qualitative Magnetic Resonance Imaging Metrics in Primary Sclerosing Cholangitis
Source: Gastro Hep Adv. 2022 Mar 30;1(3):287–95. doi: 10.1016/j.gastha.2022.01.003 (PMC11307538; doi:10.1016/j.gastha.2022.01.003)
Supplement: Table A3 [file mmc3.docx]

Supplementary Table 3. Addition of imaging covariates to liver stiffness (continuous)

|  | Model Concordance Score (95% CI) |
| --- | --- |
|  |  |
| - LS per 1 kPa - ANALI-no GAD per point | 0.88 (0.85-0.91) |
|  |  |
| - LS per 1 kPa - ANALI-GAD per point | 0.88 (0.84-0.91) |
|  |  |
| - LS per 1 kPa - Spleen length per mm | 0.89 (0.86-0.92) |
|  |  |
| - LS per 1 kPa - Spleen volume per 1000 mm^3^ | 0.91 (0.88-0.93) |
|  |  |
| - LS per 1 kPa - ANALI-no GAD per point - Spleen length per mm | 0.90 (0.88-0.93) |
|  |  |
| - LS per 1 kPa - ANALI-GAD per point - Spleen length per mm | 0.90 (0.87-0.92) |
|  |  |
| - LS per 1 kPa - ANALI-no GAD per point - Spleen volume per 1000 mm^3^ | 0.91 (0.88-0.93) |
|  |  |
| - LS per 1 kPa - ANALI-GAD per point - Spleen volume per 1000 mm^3^ | 0.90 (0.87-0.93) |

Abbreviations: LS (liver stiffness); GAD (gadolinium)
