# Supplementary material for: Comparative Performance of Quantitative and Qualitative Magnetic Resonance Imaging Metrics in Primary Sclerosing Cholangitis
Source: Gastro Hep Adv. 2022 Mar 30;1(3):287–95. doi: 10.1016/j.gastha.2022.01.003 (PMC11307538; doi:10.1016/j.gastha.2022.01.003)
Supplement: Table A4 [file mmc4.docx]

Supplementary Table 4. Bivariate analysis of imaging parameters and biochemical prognostic scores

|  | HR  (95% CI) | P  Value^a^ | Concordance  (95% CI) |
| --- | --- | --- | --- |
|  |  |  |  |
| - LS per 1 kPa - MELD | 1.55 (1.38-1.73)  1.01 (0.95-1.08) | <0.001  0.7573 | 0.89 (0.85-0.93) |
|  |  |  |  |
| - LS (> 4.70kPa=2; ≤ 4.70 kPa=0) +   ANALI-no GAD (>2 units=1; ≤2 units=0) +  Spleen Volume (>600 mm^3^=1; ≤600 mm^3^=0) per point   - MELD | 2.60 (2.06-3.28)  1.00 (0.93-1.07) | <0.001  0.9929 | 0.90 (0.86-0.94) |
|  |  |  |  |
| - LS (> 4.70kPa=2; ≤ 4.70 kPa=0) +   Spleen Volume (>600 mm^3^=1; ≤600 mm^3^=0) per point   - MELD | 3.08 (2.34-4.07)  1.01 (0.94-1.08) | <0.001  0.7608 | 0.90 (0.85-0.94) |
|  |  |  |  |
| - LS per 1 kPa - Mayo Risk Score | 1.35 (1.21-1.51)  1.85 (1.40-2.46) | <0.001  <0.001 | 0.90 (0.87-0.93) |
|  |  |  |  |
| - LS (> 4.70kPa=2; ≤ 4.70 kPa=0) +   ANALI-no GAD (>2 units=1; ≤2 units=0) +  Spleen Volume (>600 mm^3^=1; ≤600 mm^3^=0) per point   - Mayo Risk Score | 2.18 (1.78-2.67)  1.61 (1.22-2.14) | <0.001  <0.001 | 0.91 (0.89-0.94) |
|  |  |  |  |
| - LS (> 4.70kPa=2; ≤ 4.70 kPa=0) +   Spleen Volume (>600 mm^3^=1; ≤600 mm^3^=0) per point   - Mayo Risk Score | 2.62 (2.03-3.38)  1.68 (1.26-2.23) | <0.001  <0.001 | 0.90 (0.87-0.94) |
|  |  |  |  |
| - LS per 1 kPa - PRESTO | 1.40 (1.27-1.56)  10.1 (3.58-28.64) | <0.001  <0.001 | 0.91 (0.88-0.94) |
|  |  |  |  |
| - LS (> 4.70kPa=2; ≤ 4.70 kPa=0) +   ANALI-no GAD (>2 units=1; ≤2 units=0) +  Spleen Volume (>600 mm^3^=1; ≤600 mm^3^=0) per point   - PRESTO | 2.21 (1.86-2.64)  6.87 (2.69-17.54) | <0.001  <0.001 | 0.91 (0.89-0.94) |
|  |  |  |  |
| - LS (> 4.70kPa=2; ≤ 4.70 kPa=0) +   Spleen Volume (>600 mm^3^=1; ≤600 mm^3^=0) per point   - PRESTO | 2.63 (2.12-3.27)  8.34 (3.31-21.06) | <0.001  <0.001 | 0.91 (0.88-0.93) |

^a^ Cox proportional hazards regression

Abbreviations: MELD (model for end stage liver disease); LS (liver stiffness); GAD (gadolinium); PRESTO (PSC risk estimate tool)
