# Supplementary material for: Comparative Performance of Quantitative and Qualitative Magnetic Resonance Imaging Metrics in Primary Sclerosing Cholangitis
Source: Gastro Hep Adv. 2022 Mar 30;1(3):287–95. doi: 10.1016/j.gastha.2022.01.003 (PMC11307538; doi:10.1016/j.gastha.2022.01.003)
Supplement: Table A5 [file mmc5.docx]

Supplementary Table 5. Discriminative ability of each risk group within the multivariable models

| ***Dichotomous Quantitative & Qualitative***  LS (> 4.70kPa=2; ≤ 4.70 kPa=0) +  ANALI-no GAD (>2 units=1; ≤2 units=0) +  Spleen Volume (>600 mm^3^=1; ≤600 mm^3^=0)  Linear trend test, p<0.0001, HR=2.6, 95% CI=2.2-3.0 | | | | | | | | |
| --- | --- | --- | --- | --- | --- | --- | --- | --- |
|  | HR (95% CI) | P value | HR (95% CI) | P value | HR (95% CI) | P value | HR (95% CI) | P value |
| - - Score 0   - Score 1   - Score 2   - Score 3   - Score 4 | **1.0 (reference)**  9.9(3.9-24.9)  25.7(9.1-67.0)  57.7(22.9-145.6)  76.6(30.6-191.6) | <0.0001  <0.0001  <0.0001  <0.0001 | **1.0 (reference)**  2.5(1.2-5.3)  5.8(3.0-11.2)  7.7(4.1-14.7) | 0.0179  <0.0001  <0.0001 | **1.0 (reference)**  2.3(1.1-4.9)  3.1(1.5-6.5) | 0.0256  0.0025 | **1.0 (reference)**  1.3(0.7-2.5) | 0.3708 |
| ***Dichotomous Quantitative Only***  LS (> 4.70kPa=2; ≤ 4.70 kPa=0) +  Spleen Volume (>600 mm^3^=1; ≤600 mm^3^=0)  Linear trend test, p<0.0001, HR=3.2, 95% CI=2.6-3.8 | | | | | | | | |
| - - Score 0   - Score 1   - Score 2   - Score 3 | **1.0 (reference)**  8.8(4.2-18.5)  19.3(10.1-36.7)  34.9(18.4-66.2) | <0.0001  <0.0001  <0.0001 | **1.0 (reference)**  2.2(1.1-4.5)  4.0(2.0-8.0) | 0.0305  0.0001 | **1.0 (reference)**  1.8(1.01-3.2) | 0.0456 |  | |
