# Supplementary material for: Comparative Performance of Quantitative and Qualitative Magnetic Resonance Imaging Metrics in Primary Sclerosing Cholangitis
Source: Gastro Hep Adv. 2022 Mar 30;1(3):287–95. doi: 10.1016/j.gastha.2022.01.003 (PMC11307538; doi:10.1016/j.gastha.2022.01.003)
Supplement: Table A6 [file mmc6.docx]

Supplementary Table 6. Imaging predictors of hepatic decompensation, transplant, or death

|  | HR  (95% CI) | P  value^b^ | Concordance  (95% CI) |
| --- | --- | --- | --- |
| Continuous Variable^a^ |  |  |  |
| LS per 1 kPa | 1.60 (1.49-1.72) | <0.001 | 0.86 (0.83-0.90) |
|  |  |  |  |
| ANALI-GAD per 1 unit | 3.12 (2.33-4.17) | <0.001 | 0.74 (0.70-0.79) |
|  |  |  |  |
| ANALI-no GAD per 1 unit | 1.75 (1.53-2.01) | <0.001 | 0.75 (0.71-0.80) |
|  |  |  |  |
| Spleen Length per 1 mm | 1.03 (1.03-1.04) | <0.001 | 0.72 (0.67-0.78) |
|  |  |  |  |
| Spleen Volume per 1000 mm^3^ | 1.23 (1.18-1.28) | <0.001 | 0.76 (0.71-0.82) |
| Dichotomous Variables^a^ |  |  |  |
| LS >4.70 kPa | 12.12 (7.91-18.57) | <0.001 | 0.75 (0.70-0.80) |
|  |  |  |  |
| ANALI-GAD >1 | 4.84 (3.19-7.34) | <0.001 | 0.69 (0.64-0.74) |
|  |  |  |  |
| ANALI-no GAD >2 | 4.90 (3.21-7.49) | <0.001 | 0.70 (0.65-0.75) |
|  |  |  |  |
| Spleen Length >140 mm | 3.97 (2.60-6.07) | <0.001 | 0.66 (0.61-0.72) |
|  |  |  |  |
| Spleen Volume >600 (mm3) | 5.78 (3.77-8.85) | <0.001 | 0.67 (0.72-0.72) |
| Multivariable Models |  |  |  |
| *Dichotomous Quantitative & Qualitative*   - LS (> 4.70kPa=2; ≤ 4.70 kPa=0) +   ANALI-no GAD (>2 units=1; ≤2 units=0) +  Spleen Volume (>600 mm^3^=1; ≤600 mm^3^=0)^c^   - - Score 0   - Score 1   - Score 2   - Score 3   - Score 4 | (reference)  5.17 (2.65-10.11)  12.57(5.88-26.86)  27.26 (13.65-54.43)  35.11(17.67-69.79) | <0.001 | 0.85 (0.81-0.88) |
|  |  |  |  |
| *Dichotomous Quantitative Only*   - LS (> 4.70kPa=2; ≤ 4.70 kPa=0) +   Spleen Volume (>600 mm^3^=1; ≤600 mm^3^=0)^c^   - - Score 0   - Score 1   - Score 2   - Score 3 | (reference)  5.14 (2.62-10.12)  13.14(7.62-22.67)  21.91(12.53-38.31) | <0.001 | 0.80 (0.76-0.85) |
|  |  |  |  |

^a^ Unadjusted variables

^b^ Cox proportional hazards regression

Abbreviations: LS (liver stiffness); GAD (gadolinium).
